# Comparison of olecranon osteotomy and paratricipital approach in distal humerus intra-articular fracture: A systematic review and meta-analysis

**DOI:** 10.1097/MD.0000000000030289

**Published:** 2022-08-26

**Authors:** Ho-Seung Jeong, Jae Young Yang, Seung Jun Jeon, Hyun-Chul Shon, Jong-Keon Oh, Eic Ju Lim

**Affiliations:** a Department of Orthopaedic Surgery, Chungbuk National University Hospital, Chungbuk National University College of Medicine, Cheongju, Republic of Korea; b Department of Orthopedic Surgery, Korea University Guro Hospital, Seoul, Republic of Korea.

**Keywords:** complication, distal humerus fracture, elbow function, olecranon osteotomy, operation time, paratricipital

## Abstract

Surgical outcomes of the paratricipital approach (PT) and olecranon osteotomy (OO) for the treatment of distal humerus intra-articular fracture have been reported. However, there is no consensus regarding which approach is better in terms of functional outcomes and complications. This study aimed to compare the operation time, functional outcomes, and complications of OO and PT.

**Methods:**

The databases of MEDLINE, Embase, and Cochrane Library were systematically searched for studies published before June 1, 2021. We performed synthetic analysis of the operation time, functional outcomes, and incidence of complication after the conduct of OO group or PT group in patients with distal humerus intra-articular fractures.

**Results:**

Five studies were included representing a total of 243 patients who underwent surgery for distal humerus intra-articular fractures. A pooled analysis showed that there was a longer operation time in the OO group compared with the PT group (mean difference [MD] = 13.32, 95% CI: 3.78–22.87; *P* = .006). There was no significant difference between the functional outcomes of the OO and PT groups (elbow flexion: MD = 2.4, 95% CI: −0.82 to 5.79, *P* = .14; elbow extension: MD = 0.36, 95% CI: −2.20 to 2.92, *P* = .78; elbow arc of motion: MD = 0.40, 95% CI: −4.05 to 4.84, *P* = .86; Mayo Elbow Performance score: MD = −1.37, 95% CI: −4.73 to 1.98, *P* = .42). The incidence of infection was significantly higher in the OO group compared with that of the PT group (odds ratio [OR] = 3.82, 95% CI: 1.03–14.16, *P* = .04). There was no significant difference between the 2 groups in terms of the heterotopic ossification and ulnar neuropathy (OR = 1.85, 95% CI: 0.51–6.71, *P* = .35 and OR = 2.74, 95% CI: 0.60–12.48, *P* = .19, respectively).

**Conclusions:**

Since the choice of surgical approach does not influence outcomes, surgeons can base their choice of approach on the basis of their own experience and familiarity with the procedure and the need to visualize the entire articular surface in complex intra-articular fracture patterns.

## 1. Introduction

Distal humerus fractures are relatively uncommon, accounting for 8% of humeral fractures and 2% of elbow fractures in the adult population.^[1,2]^ Intra-articular fractures are more likely to result in complications such as post-traumatic osteoarthritis, heterotopic ossification, and joint stiffness.^[3]^ To achieve satisfactory outcomes, anatomical reduction, stable fixation, and early range of motion are essential. Standard practice for fixation of distal humerus intra-articular fracture is to apply 2 plates (one medial, 1 lateral) to provide a bicolumnar support construct.^[4]^

**Figure 1.**
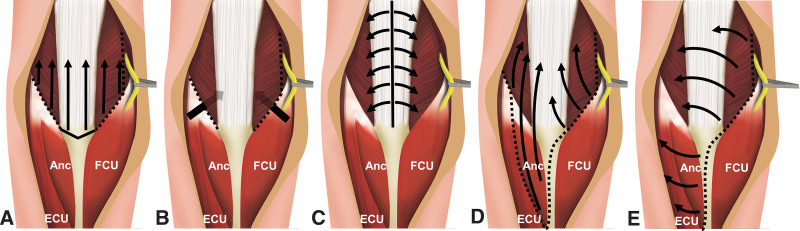
Schematic illustration of the posterior approaches to the distal humerus. (A) Olecranon osteotomy, (B) paratricipital approach, (C) triceps-splitting approach, (D) triceps-reflecting anconeus pedicle approach, and (E) Bryan-Morrey approach. Anc = ancenous, ECU = extensor carpi ulnaris, FCU = flexor carpi ulnaris.

With development of the fixation technique, several approaches were introduced to reduce intra-articular fracture (Fig. 1). Olecranon osteotomy (OO) is a conventional approach that was first described by Russell MacAusland^[5]^ and has been reported to provide maximum exposure of the distal articular surface.^[6]^ The triceps-reflecting anconeus pedicle approach was presented by O’Driscoll,^[7]^ in which the anconeus and triceps were reflected proximally. The triceps-sparing approach was reported by Bryan-Morrey, which focused on total elbow arthroplasty.^[8]^ The application of this approach in the treatment of distal humerus fractures has been reported in several studies,^[9–11]^ including systematic reviews that have compared triceps-sparing approach with OO.^[12]^

The paratricipital approach (PT), introduced by Alonso-Llames in 1972^[13]^ for the treatment of supracondylar fractures in children and modified by Schildhauser et al^[14]^ for exposure of the articular surface of the distal humerus, has been investigated relatively recently. This approach offers the advantage of sparing the insertion of the triceps, which results in preservation of the extensor mechanism, when compared with other approaches. Several reports have compared surgical outcomes between OO and the PT^[15,16]^; however, there is no consensus regarding which approach is better in terms of functional outcomes and complications. To the best of our knowledge, there has been no large-scale synthetic study on this topic. The purpose of this study was to compare OO with the PT in terms of operation time, functional outcome, and complications.

## 2. Materials and Methods

This study was performed in accordance with the Cochrane Review and Preferred Reporting Items for Systematic Review and Meta-Analysis Protocols guidelines.^[17,18]^ Although human participants were involved in present study, ethical approval or informed consent from the participants was not required because all data were based on previously published studies that were analyzed anonymously without any potential harm to the participants.

### 2.1. Literature search

Based on these guidelines, we searched the MEDLINE, Embase, and Cochrane Library databases for comparative studies that had investigated the surgical outcomes following each approach in the treatment of distal humerus intra-articular fractures. The search was performed for articles from inception to June 1, 2021, using an a priori search strategy. Search terms included synonyms and related terms for distal humerus fracture and approach as follows: (“distal humer*” OR “humerus intercondylar” OR “humeral intercondylar”) AND (fractur*) AND (“olecranon osteotomy”) AND (“paratricipital” OR “paratriceps” OR (“triceps” AND “sparing”) OR (“olecranon” AND “sparing)). There were no restrictions on language, publication year, and type of publication. After the initial electronic search, relevant studies and their bibliographies were manually searched.

### 2.2. Study selection

Based on the titles and abstracts of the studies obtained from the search, 2 board-certified orthopedic surgeons with orthopedic trauma fellowship, independently selected studies for full-text review. If the abstract provided insufficient data to make a decision, the full article was reviewed.

In this systematic review, we included studies that directly compared surgical outcomes between OO and PT described by Alonso-Llames^[13]^ and Schildhauser et al^[14]^ in the treatment of distal humerus intra-articular fractures (AO/OTA 13C). We excluded biomechanical and cadaveric studies, technical notes, letters to the editor, expert opinions, review articles, meta-analyses, conference abstracts, and case reports.

At each stage of study selection, the κ-value was calculated to determine inter-reviewer agreement regarding study selection. Agreement between reviewers was correlated a priori with κ-values as follows: κ = 1 corresponded to “perfect” agreement; 1.0 > κ ≥ 0.8 to “almost perfect” agreement; 0.8 > κ ≥ 0.6 to “substantial” agreement; 0.6 > κ ≥ 0.4 to “moderate” agreement; 0.4 > κ ≥ 0.2 to “fair” agreement; and κ < 0.2 to “slight” agreement. Disagreements at each stage were resolved by consensus between the 2 investigators, or by discussion with a third investigator, who was a board-certified orthopedic surgeon, when the consensus could not be reached.

### 2.3. Data extraction

For the qualitative synthesis, we extracted data on patient demographics including the number of patients, patient age, sex, type of fracture (AO/OTA^[19]^), and the number of patients with AO/OTA 13-C3. Indication of the approach and follow-up period were also extracted. Noncomparable complications (e.g., nonunion of OO and symptomatic prominence of OO) between groups were extracted using a standardized form.

For the meta-analysis, we extracted the data of operation time, functional outcome including range of motion of the elbow (elbow flexion, extension, and arc of motion) and Mayo Elbow Performance score (MEPS), operation time, and complications (e.g., heterotopic ossification, infection, and ulnar neuropathy).

For all of the data extraction, the same 2 board-certified orthopedic surgeons, who participated in the study selection, independently recorded the data from each enrolled study. Disagreements between the reviewers were resolved by discussion between the 2 investigators.

### 2.4. Methodological quality assessment

The methodological quality of the included studies was evaluated using the Newcastle-Ottawa scale.^[20]^ Two independent reviewers performed a quality assessment and resolved disagreements through discussion.

### 2.5. Data synthesis and statistical analyses

The main outcomes of the present meta-analysis were operation time, functional outcome, complications of OO group, and PT group. The following comparisons were included as functional outcomes: elbow flexion, elbow extension, elbow arc of motion, and MEPS. Complications included: heterotopic ossification, infection, nonunion of main fracture, and ulnar neuropathy.

For all comparisons, the continuous data were analyzed using mean differences (MD) with 95% confidence intervals (CI). Heterogeneity was assessed using the *I*^2^ statistic, in which 25%, 50%, and 75% were considered as low, moderate, and high heterogeneity, respectively. Forest plots were used to show the outcomes, pooled estimates of effects, and overall summary effect of each study. Statistical significance was set at *P*-value<.05. All data were pooled using a random-effects model, which was recommended previously to avoid overestimation of the study results, especially in the medicine field.^[21]^ We did not perform the test for publication bias because the evaluation is typically performed only when at least 10 studies are included in the meta-analysis.^[22]^ All statistical analyses were performed using Review Manager (RevMan), version 5.3 (The Nordic Cochrane Centre, The Cochrane Collaboration, Copenhagen, Denmark).

## 3. Results

### 3.1. Study identification

**Figure 2.**
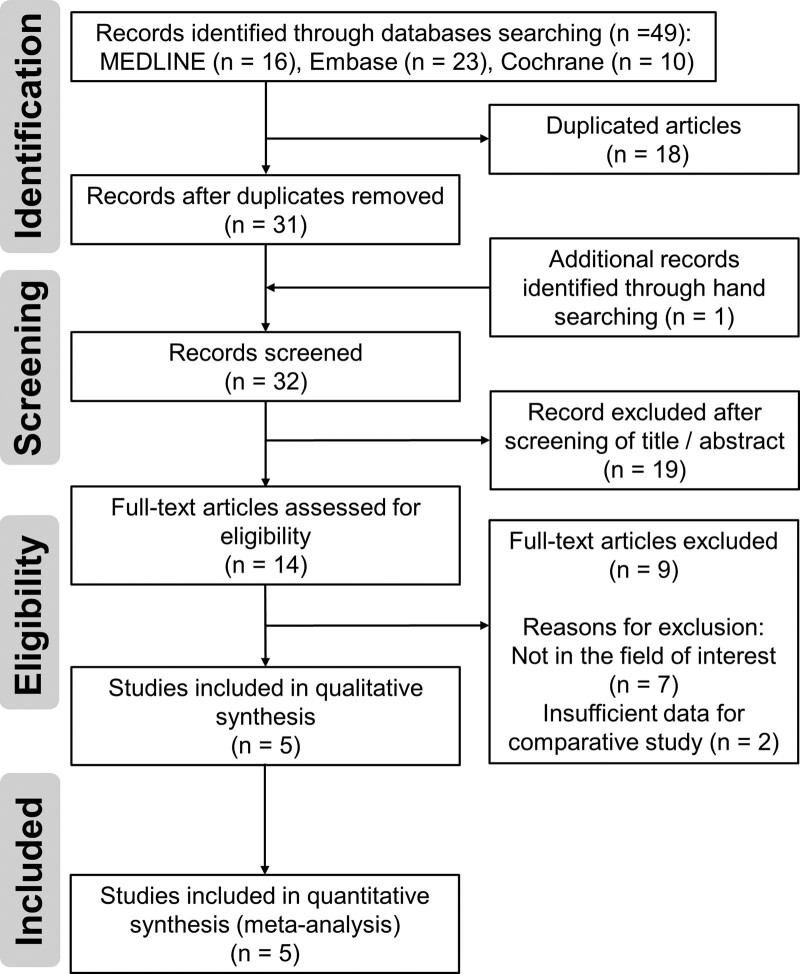
Preferred Reporting Items for Systematic Reviews and Meta-analyses flow diagram describing the process of the literature selection.

The details of the study identification and selection process are summarized in Figure 2. The initial electronic literature search yielded 49 articles. After exclusion of 18 duplicates and addition of 1 article identified by manual searching, 32 studies were screened; 18 studies were excluded after their titles and abstracts were reviewed, and 9 studies were excluded after full-text review. Eventually, 5 studies were included in the meta-analysis.^[15,16,23–25]^ Agreement between the reviewers on study selection was “almost perfect” at the title and abstract review stage (κ = 0.875), and the full-text review stage (κ = 0.851).

### 3.2. Study characteristics and qualitative synthesis

Of the 5 studies included in the meta-analysis, 4 were retrospective comparative studies^[15,23–25]^ and 1 was a prospective comparative study.^[16]^ There were no randomized controlled trials comparing OO and the PT. In the current study, the 5 studies included 243 patients who underwent surgery for distal humerus intra-articular fracture. OO was performed in 123 patients, and the PT in 120 patients. The average age of the patients was 37.5 to 51.8 years in 4 studies.^[15,16,23,24]^ One study included patients older than 65 years where the average patient age was 78.2 years old.^[25]^ Three studies included all types of distal humerus intra-articular fractures (AO/OTA 13-C1, 2, 3).^[16,24,25]^ One study included only AO/OTA 13-C3 fractures,^[23]^ and 1 included AO/OTA 13-C2 fractures.^[15]^ Two studies presented specific number of patients in each fracture type.^[16,24]^ The OO approach demonstrated higher ratio of AO/OTA 13-C3 than the PT in both studies.

Four studies presented the indication for selection of the approach,^[15,16,24,25]^ of which 2 selected the approach based on surgeon preference.^[16]^ One study used OO for the first half of the study period and the PT for the second half of the study period.^[24]^ The mean follow-up period ranged from 13.2 to 46.4 months. Further details of patient demographics are described in Table 1.

**Table 1:** The demographic data and the indications for the approaches.

	Study design	Patient number		Age (yr)	Male sex (%)	AO/OTA classification	Number of AO/OTA 13-C3		Indication for the approach	Follow-up period (mo)
		PT	OO				PT	OO		
Zhang et al (2018)	RCS	17	19	40.5	26 (72%)	13 C3	17/17 (100%)	19/19 (100%)	N/A	22.3
Jacko et al (2019)	RCS	18	22	51.8	12 (30%)	13 C1, 2, 3	4/18 (22%)*	13/22 (59%)*	Surgery period	13.2
Singh et al (2019)	PCS	27	24	40.5	29 (57%)	13 C1, 2, 3	6/27 (22%)†	12/24 (50%)†	Expertise of trauma unit that patients admitted into	24.3
Ansari et al (2020)	RCS	32	28	37.5	33 (55%)	C2	0/28 (0%)	0/33 (0%)	Comminution, type, experience, and preference	46.4
Kaiser et al (2020)	RCS	26	30	78.2	16 (29%)	13 C1, 2, 3	N/A	N/A	Fracture stability, skin integrity, vascular injury, trochlear comminution, and medical comorbidities	15.2

Comparison for patient number of C3 presented significant differences (*P = .019 and †*P* = .046).

N/A = not available, OO = olecranon osteotomy, PCS = prospective comparative study, PT = paratricipital approach, RCS = retrospective comparative study.

### 3.3. Risk of bias assessment

There were no randomized controlled trials; non-randomized comparative studies were found. Of the 5 comparative studies included, 1 was graded as 5, 2 as 6, 1 as 7, and 1 as 8, as per the Newcastle-Ottawa scale (Table 2). The major source of bias was non-comparability of the study group, especially regarding different criteria for selecting approaches.

**Table 2:** Assessment of the included cohort studies using the New Castle Ottawa Scale.

Author	Selection of cohort (4)	Comparability of cohort (2)	Assessment of outcome (3)	Total score
Zhang et al	★★★	★	★★	6
Jacko et al	★★★	★	★★	6
Singh et al	★★★	★	★★★	7
Ansari et al	★★★★	★	★★★	8
Kaiser et al	★★★	–	★★	5

#### 3.3.1. Operation time.

**Figure 3.**
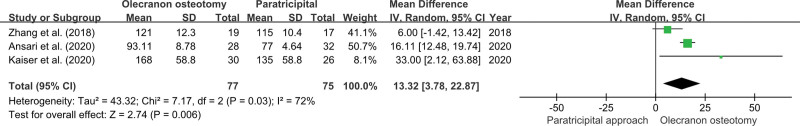
Results of an aggregate analysis that compares the operation times between olecranon osteotomy and the paratricipital approach. CI = confidence interval, SD = standard deviation.

Three studies compared the *operation time* between OO (n = 77) and PT (n = 75).^[15,23,25]^ The pooled data showed that the MD was 13.32 minutes longer in the OO group than in the PT group and was statistically significant (95% CI: 3.78–22.87; *P* = .006). The heterogeneity was considered high (*I*^2^ = 72%). A Forest plot is shown in Figure 3.

#### 3.3.2. Function of the elbow.

**Figure 4.**
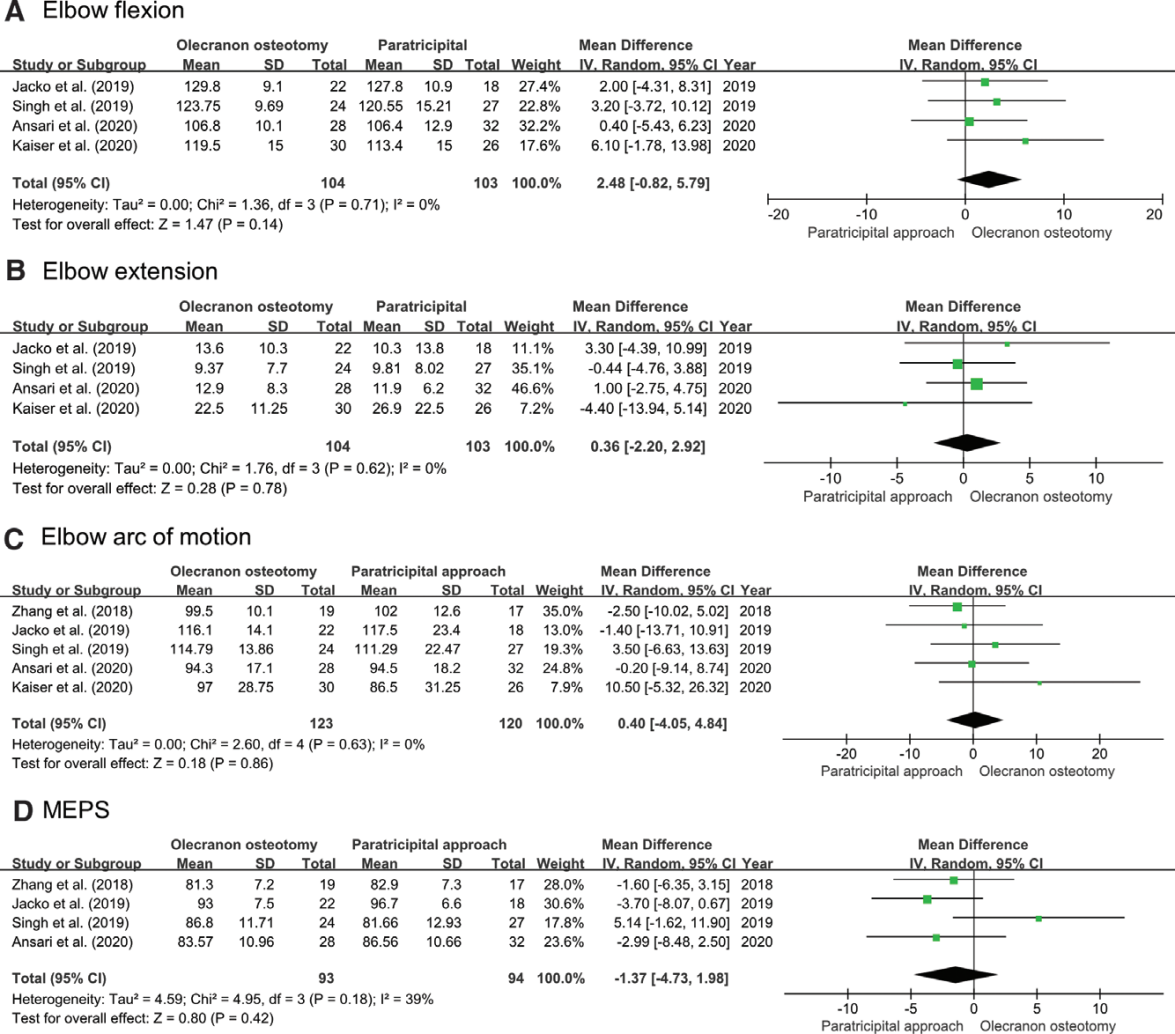
Results of an aggregate analysis that compares the functional outcomes between olecranon osteotomy and the paratricipital approach: (A) elbow flexion, (B) elbow extension, (C) elbow arc of motion, and (D) MEPS. CI = confidence interval, MEPS = Mayo Elbow Performance score, SD = standard deviation.

Four studies compared *elbow flexion* between OO (n = 104) and PT (n = 103).^[15,16,24,25]^ The pooled data showed that there was no statistical difference between the 2 approaches (MD = 2.48; 95% CI: −0.82 to 5.79; *P* = .14; *I*^2^ = 0%). Four studies compared *elbow extension* between OO (n = 104) and PT (n = 103).^[15,16,24,25]^ The pooled data showed no significant difference between the 2 approaches (MD = 0.36; 95% CI: −2.20 to 2.92; *P* = .78; *I*^2^ = 0%). All 5 studies compared the *elbow arc of motion* between OO (n = 123) and PT (n = 120).^[15,16,23–25]^ The pooled data showed that there was no statistical difference between the 2 approaches (MD = 0.40; 95% CI: −4.05 to 4.84; *P* = .86; *I*^2^ = 0%). Four studies compared *MEPS* between OO (n = 93) and the PT (n = 94).^[15,16,23,24]^ The pooled data showed that there was no statistical difference between the 2 approaches (MD = −1.37; 95% CI: −4.73 to 1.98; *P* = .42; *I*^2^ = 39%). A Forest plot is shown in Figure 4.

#### 3.3.3. Complications.

**Figure 5.**
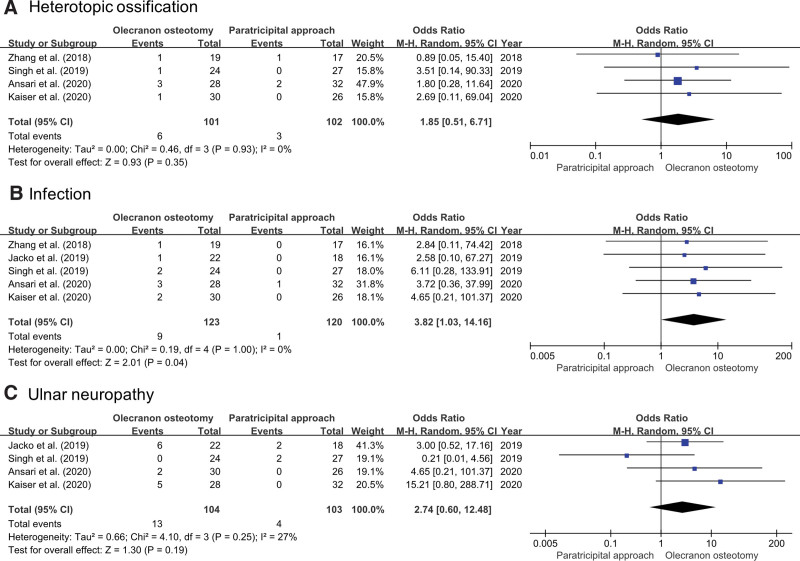
Results of an aggregate analysis that compares the complications between olecranon osteotomy and the paratricipital approach: (A) heterotopic ossification, (B) infection, and (C) ulnar neuropathy. CI = confidence interval.

Four studies compared *heterotopic ossification* between OO (n = 101) and the PT (n = 102).^[15,16,23,25]^ A pooled analysis revealed no significant differences in the incidence of heterotopic ossification between the groups (odds ratio [OR], 1.85; 95% CI, 0.51–6.71; *P* = .35; *I*^2^ = 0%). All 5 studies compared *infection rates* between OO (n = 123) and the PT (n = 120).^[15,16,23–25]^ The pooled analysis revealed that the infection rate was significantly higher in OO than that in the PT (OR = 3.82; 95% CI, 1.03–14.16; *P* = .04). The heterogeneity was considered low (*I*^2^ = 0%). Four studies compared *ulnar neuropathy* between OO (n = 104) and the PT (n = 103).^[15,16,24,25]^ A pooled analysis revealed no significant differences in ulnar neuropathy between the groups (OR, 2.74; 95% CI, 0.60–12.48; *P* = .19; *I*^2^ = 58%). A Forest plot is shown in Figure 5.

*The nonunion rate of the main fracture* cannot be analyzed because only 1 study presented comparable data regarding nonunion.^[24]^ Four studies reported no nonunion of the main fracture. Two patients in the included 5 studies demonstrated *nonunion of OO* (2/123, 2%). Eight patients presented *symptomatic prominence of OO fixation* in 4 studies^[15,16,24,25]^ (8/104, 8%). Details are presented in Table 3.

**Table 3:** Other complications.

	Nonuion of fracture		Nonunion of OO	Symptomatic prominence of OO fixation
	PT	OO		
Zhang et al (2018)	0 (0%)	0 (0%)	0 (0%)	N/A
Jacko et al (2019)	0 (0%)	1 (5%)	1 (5%)	0 (0%)
Singh et al (2019)	0 (0%)	0 (0%)	0 (0%)	4 (17%)
Ansari et al (2020)	0 (0%)	0 (0%)	1 (3%)	3 (11%)
Kaiser et al (2020)	0 (0%)	0 (0%)	0 (0%)	1 (3%)
Total	0/120 (0%)	1/123 (1%)	2/123 (2%)	8/104 (8%)

OO = olecranon osteotomy, PT = paratricipital approach.

## 4. Discussion

This qualitative synthesis demonstrated that there was no difference in the functional outcome between the 2 groups. However, increased operation time and postoperative infection in OO group, although OO group had more proportion of AO/OTA 13-C3 fracture type than PT group in this synthetic study. We believe that the proper approach should be selected based on the fracture type, rather than using a single consistent approach for treatment of distal humerus intra-articular fracture.

In our systematic review, we observed several interesting findings. First, there was a lack of high-quality data comparing surgical approaches. Although 5 comparative studies were included, methodological flaws were observed especially as selection bias. We could not identify any randomized controlled trials. Therefore, the results of present study should be interpreted carefully. Second, we encountered mixed use of the terminology “triceps-sparing” which was used to describe the triceps-sparing approach by Bryan and Morrey^[8]^ and the PT by Alonso-Llames^[13]^ and Schildhauer et al^[14]^ (Table 4). The Bryan-Morrey approach dissects the insertion of the triceps muscle with continuity on the radial side, and the PT preserves the insertion of the triceps muscle without detachment of its insertion (Fig. 1). We focused on the PT with a detailed review of the references and description of the approach in each study.

**Table 4:** Terminologies and references of the approaches.

	Terminology of approach	Reference approach
Liu et al (2009)	Triceps sparing	No specific reference for approach
Chen et al (2011)	Triceps-sparing	Bryan and Morrey
Zhang et al (2014)	Triceps-sparing	Bryan and Morrey
Khalid et al (2015)	Triceps sparing	Bryan and Morrey
Zhang et al (2018)	Paratricipital	No specific reference for approach
Jacko et al (2019)	Paratricipital (triceps on and triceps sparing)	Alonso-LlamesSchildhauer
Singh et al (2019)	Paratricipital	Schildhauer
Ansari et al (2020)	Triceps sparing	Schildhauer
Kaiser et al (2020)	Limited fixation (L-ORIF) Paratricipital approach	Alonso-Llames

There were no significant differences in the functional outcomes, including elbow flexion, extension, arc of motion, and MEPS, between OO and PT groups. Stiffness after distal humerus fracture has been reported to be mainly affected by fracture severity.^[26]^ Sharma et al^[27]^ performed a synthetic study for functional outcomes between OO and Bryan and Morrey or triceps-splitting approaches and concluded that there were no differences in the functional outcomes between the approaches. In distal humerus fracture, functional outcome seems to be influenced by fracture severity rather than surgical approach.

In the present qualitative synthesis study, incidence of heterotopic ossification did not show any significant differences. In OO, additional muscle damage is limited usually to the proximal portion of the anconeus by surgical dissection, which does not affect the incidence of heterotopic ossification. There were no significant differences in either transient or persistent ulnar neuropathy. In general, both approaches needed dissection and preservation of the ulnar nerve during the surgery; however, detailed description of the ulnar nerve during surgery was insufficient in the included studies. There was a controversy about the transposition of the ulnar nerve during distal humerus fracture surgery. Gofton et al^[28]^ strongly supported routine transposition of the ulnar nerve, which showed a 0% rate of postoperative ulnar neuropathy in patients who underwent transposition. In contrast, Chen et al presented 3.7 times higher incidence of ulnar neuropathy in patients who underwent transposition.^[29]^ Wilson et al^[30]^ reported that addition of OO to the PT did not increase ulnar neuropathy; however, this study was excluded from this meta-analysis at the final full-text review stage because it included AO/OTA 13A, B fractures. Further research is needed to elucidate the strategy for appropriate handling of the ulnar nerve and to understand the effect of surgical approach on the ulnar nerve.

In the present study, the OO group demonstrated more surgical time and higher infection rate than the PT group, suggesting that OO is not a minor procedure. There were concerns about osteotomy, reduction, and refixation in OO. Coles et al^[31]^ reported that approximately 1/3 of patients who underwent OO underwent removal of the olecranon fixation and 8 of 104 (8%) patients included in present study complained of symptomatic prominence of the OO fixation. However, despite those concerns, there can be situations where OO is preferred. For example, full exposure of the articular surface is difficult using the PT without OO. Cho et al^[32]^ presented 20 mm of inaccessible central articular segment (30% of transepicondylar width) for PT in a cadaveric study. In addition, manipulation of the articular fragment as well as exposure needs adequate space around the articular surface. If fracture type is considered feasible with PT, PT could be tried; however, the surgeon should be prepared for additional OO in inaccessible and irreducible cases.

The current meta-analysis has several limitations. First, the number of included studies is relatively small. Even after a systematic search with no restrictions on language and publication year, we identified only 5 suitable studies for quantitative synthesis. Nevertheless, considering that our study is the first-meta-analysis that provides a comparative overview of this topic, we believe that the results were meaningful. Second, all the studies included in the meta-analysis were retrospective in nature. The indication of OO or the PT was not specified; hence, this could have led to a selection bias. We discussed the results of the present study under consideration of these biases. Third, due to limited available data, we could not conduct meta-analysis of the reduction status. The reduction status could be directly affected by the approach, and malreduction could lead to poor radiological and functional outcomes. Prospective studies including more variables are required to analyze these issues more clearly.

## 5. Conclusion

In present study, there was no difference in the postoperative functional outcomes between OO and the PT; however, the latter demonstrated shorter surgical time and lower infection rate than the former. Since the choice of surgical approach does not influence outcomes, surgeons can base their choice of approach on the basis of their own experience and familiarity with the procedure and the need to visualize the entire articular surface in complex intra-articular fracture patterns.

## Author contributions

**Conceptualization:** Eic Ju Lim.

**Data curation:** Jae Young Yang, Hyun-Chul Shon.

**Formal analysis:** Jae Young Yang.

**Methodology:** Jae Young Yang.

**Validation:** Hyun-Chul Shon.

**Visualization:** Eic Ju Lim.

**Writing – original draft:** Ho-Seung Jeong, Eic Ju Lim.

**Writing – review & editing:** Ho-Seung Jeong, Seong Jun Jeon, Jong-Keon Oh, Eic Ju Lim.
